# The potential dysfunction of otolith organs in patients after mumps infection

**DOI:** 10.1371/journal.pone.0181907

**Published:** 2017-07-26

**Authors:** Yu-Juan Zhou, Jing Yu, Yong-Zhen Wu, Liang Tian, Zhao Han, Jing Wang, Fang-Lu Chi

**Affiliations:** 1 Department of Otology and Skull Base Surgery, Eye Ear Nose & Throat Hospital, Fudan University, Shanghai, China; 2 Shanghai Auditory Medical Center, Shanghai, China; 3 Key Laboratory of Hearing Science, Ministry of Health, Shanghai, China; National Taiwan University Hospital, TAIWAN

## Abstract

**Objective:**

To investigate the relationship between mumps and the extent of hearing impairment and otolith organ damage.

**Methods:**

A total of 27 patients with unilateral hearing impairment following mumps were enrolled. The degrees of hearing loss and otolith organ damage were confirmed by audiometric and vestibular evoked myogenic potential [VEMP] tests. All the results were compared and analyzed using Stata 13.0 software for Windows.

**Results:**

The VEMP thresholds of the affected ears were significantly higher than those of the unaffected ears in both tests (cervical VEMP [cVEMP] test and ocular VEMP [oVEMP] test; p = 0.000 and 0.001, respectively). The mean cVEMP and oVEMP threshold values of the affected ears with hearing impairment for ≤10 years were significantly lower than those of affected ears with hearing impairment for >10 years [p = 0.009 and 0.004, respectively].

**Conclusions:**

Deafness resulting from mumps is usually profound and permanent, which indicates severe damage to the cochlea due to the disease. The functions of otolith organs in the vestibular system are also impaired. Over time, the function of the otolith organs or their neural pathway may suffer secondary damage.

## Introduction

Mumps, an acute respiratory infectious disease, is caused by the mumps virus, which is a single-stranded RNA virus of the family Paramyxoviridae. Mumps is easily transmitted among humans by oropharyngeal secretions. It is, therefore, endemic worldwide and has a high risk of outbreaks in schools and childcare facilities, as children and adolescents are highly susceptible to mumps [1]. Between 2008 and 2011, a total of about 909,087 cases of mumps were reported in China, with an annual incidence rate of 22.8/100,000 and a male to female ratio of 1.62:1. Up to 81.8% of these cases were reported in children between the ages of 3 and 14 years, with an overwhelming majority of cases reported in childcare institutions and primary schools [2]. The morbidity and mortality of mumps can be reduced by routine vaccination. However, the re-emergence of mumps and its increased incidence has been reported in several countries, especially the developed countries [3]. The resurgence of mumps might be due to mutations in the mumps virus and inadequate immunization of susceptible groups of people [4].

The most prominent manifestation of mumps is unilateral or bilateral painful enlargement of the parotid glands [5]. However, about a third of unvaccinated patients with mumps might have mild or silent symptoms [6]. Following an incubation period, infection with the mumps virus might give rise to various symptoms, including fever, orchitis, meningitis, and hearing loss [7]. In a recent investigation of 3130 mumps cases, Zamir et al. reported that 13.4% of the patients exhibited systemic symptoms and/or complications [8].

Serious complications of mumps infection include hearing loss, aseptic meningitis, and encephalitis, and the existence of the latter two might increase the risk of sensorineural hearing loss (SNHL) [9–10]. It has been reported that the incidence of deafness in mumps is 0.5–5.0 per 100,000 cases; this rate might be higher in places where mumps is endemic [11]. Although mumps is an apparently mild disease, it can cause hearing impairment. Flu-like symptoms tend to present 3–7 days after mumps infection, and ipsilateral or bilateral sensorineural deafness might suddenly occur [12]. The severity of mumps infection and the incidence of severe hearing loss due to mumps are not associated [13]. However, it should be stressed that vaccination can minimize the incidence of hearing loss. During a mumps outbreak in the United States in 2006, relatively few cases of deafness were reported after vaccination [14].

Besides possibly causing sudden unilateral hearing loss, mumps can also result in vestibular dysfunction. Several patients with hearing impairment exhibit vestibular pathology in the affected ear after mumps infection [15]. Vestibular evoked myogenic potential [VEMP] tests have conventionally been used for the evaluation of otolithic function in patients with vestibular disorders [16]. Cervical VEMP (cVEMP) values are considered to reflect the functions of the saccular and inferior vestibular nerve input pathways, and it has been shown that saccular neurons have a strong projection to the neck muscles, but only a weak projection to the oculomotor system [17]. Ocular VEMP (oVEMP) values are deemed to reflect the function of the utricle and the superior vestibular nerve input pathway; it has been shown that utricular neurons have a strong projection to the oblique muscle of the lower eyelid, and the oVEMP is generated by the activation of the utricular afferents and mediated by the otolith-ocular reflex pathway [18].

The probable relationship between vestibular disorders and mumps had been mentioned in some studies, but detailed studies are rare. In this study, we attempted to evaluate the extent of hearing impairment and otolith organ damage after mumps infection further.

## Materials and methods

### Subjects

This study was approved by Ethical Board of Eye Ear Nose and Throat Hospital, Fudan University, and all of the patients provided written informed consent. This retrospective study included 27 patients who visited the outpatient Hearing and Vertigo Clinic in the Eye Ear Nose & Throat Hospital of Fudan University from May 2014 to January 2016. The 27 patients, 19 males and 8 females, were aged 5 to 51 years, with a mean age (± standard deviation) of 18.81 ± 11.65 years. All patients whose unilateral hearing impairment occurred within 1 week after definite mumps infection were enrolled and underwent audiometry and VEMP tests to confirm the degree of permanent lesions. The included patients exhibited neither any neurological or cardiovascular diseases, nor other ear diseases, such as otitis media, tympanosclerosis, or Meniere’s disease. The intactness of each eardrum was confirmed by otoscopy, and the VEMP values of 54 ears (27 patients) were analyzed.

### Vestibular evoked myogenic potential test

In the VEMP test, the patients were placed supine in a sound-proof examination room maintained at a temperature of 24–26°C. An auditory stimulus was applied using air-conducted sound [ACS] with a short tone burst (1 ms rise/fall time and 2 ms plateau time) at 500 Hz. The electromyography signals were amplified by a Bio-Logic Navigator PRO system (Bio-logic Auditory Evoked Potential Ver. 7.0.0 software, Bio-Logic Systems Corp, Mundelein, IL), and the band pass-filtered sounds between 10–1500 Hz. In total, 120 stimuli were applied to each ear. The sound stimuli (500 Hz) were presented via calibrated headphones. The starting intensity was 95 dB nHL, which was decreased in decrements of 5 dB nHL until the VEMP response was abolished. This process was repeated three times. The intensity of the last characteristic waveform of VEMP was defined as the response threshold.

### Cervical vestibular evoked myogenic potential test

An active electrode on the middle of the sternocleidomastoid muscle (SCM), with a reference electrode on the lower part of the suprasternal fossa and a ground reference on the middle of the forehead. The cVEMP waveforms were recorded after a total of 120 responses, induced by the stimuli of ACS with a 500-Hz short tone burst transmitted via the insert headphones, were averaged. The presence of cVEMP was registered when the amplitude of the first positive-negative-positive peak (P1-N1-P2) was recorded. The absence of cVEMP was recorded in cases where the waveforms could not be recognized or were not repeatable. Before placing the electrodes, the skin was scrubbed off in order to decrease the electrode impedance to < 5 KΩ. During the test, patients were instructed to elevate their heads to activate the SCM electrode when they heard a sound on the calibrated insert headphones.

### Ocular vestibular evoked myogenic potential test

In the oVEMP test, an active electrode was placed about 1 cm below the center of the inferior eyelid of the right eye contrary to the side of sound stimulation, with a reference electrode 2cm below the active electrode and a ground reference on the middle of the forehead. The oVEMP waveforms were recorded after a total of 120 responses, induced by ACS stimuli with a 500-Hz short tone burst transmitted via the insert headphones, were averaged. The presence of oVEMP was registered when the peaks of the first negative and positive biphasic wave, designated as N1 and P1, respectively, were recorded. The absence of oVEMP was recorded in cases where the waveforms could not be recognized or were not repeatable. Before placing the electrodes, the skin was scrubbed off in order to decrease the electrode impedance to <5 KΩ. During the test, patients were instructed to fix their gaze on a point about 30° above the horizontal line when they heard a sound on the calibrated insert headphones.

### Statistical analysis

Statistical analysis was performed using the Stata 13.0 software for Windows. Descriptive statistical methods were used to describe the frequencies of the evaluated variables. Chi-square tests were used to compare the response rates of the VEMPs. Wilcoxon’s signed-rank test was used to compare the threshold values of the VEMPs between the affected and unaffected ears. A two-sample Wilcoxon rank-sum [Mann-Whitney] test was performed to evaluate the difference in the degree of damage to otolith organs between patients with hearing loss lasting ≤ 10 years and those with hearing loss lasting > 10 years. Values of p < 0.05 were considered statistically significant.

## Results

### Extent of unilateral hearing impairment after mumps

The average age of the 27 patients at the time of infection with mumps was 8.56 ± 4.03 years. All of the patients exhibited unilateral hearing impairment after mumps. While 13 patients suffered hearing impairment in the right ear, 14 were affected in the left ear. Among the affected ears, 21 (77.8%) exhibited no response during audiometric examination, but six (22.2%) exhibited profound sensorineural hearing loss (Table 1). All 27 unaffected ears (100%) exhibited normal hearing levels.

**Table 1:** Audiometry results of the 27 patients.

Audiometry test [dB nHL]	Affected ears	Unaffected ears
≤ 25	0	27 [100%]
90–130	6 [22.2%]	0
No response	21 [77.8%]	0

Data are presented as number [percentage].

### The symptoms and syndromes of the vestibular system

Only one patient suffered from vertigo and exhibited mild abnormalities in the caloric test and sensory organization test. The other 26 patients did not have any complaint of dizziness and imbalance or any syndrome of vestibular dysfunction.

### Differences in VEMPS between the affected and unaffected ears

**Fig 1:**
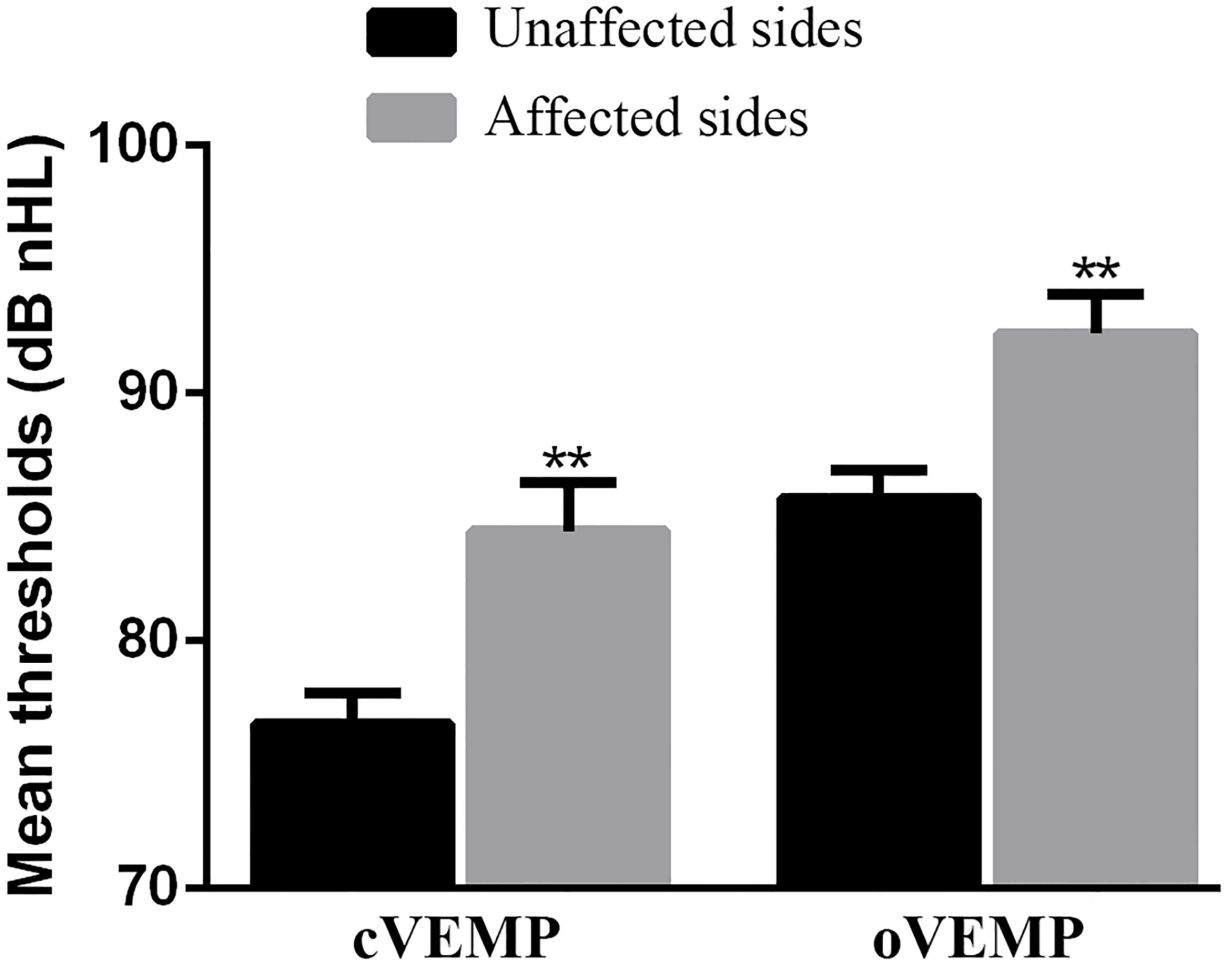
Differences in mean threshold values between the unaffected and affected ears of patients with unilateral hearing impairment after mumps. The columns of the histograms represent the mean values of thresholds and the standard error bars are marked in the histograms. **p < 0.01. cVEMP, cervical vestibular evoked myogenic potential; oVEMP, ocular vestibular evoked myogenic potential.

In the cVEMP test, 22 patients (81.48%) exhibited a bilateral response, five (18.52%) exhibited no response in the affected ear. The response rate of the affected ears was lower than that of the unaffected ears (p = 0.019; Table 2). While 18 patients (66.67%) exhibited higher cVEMP threshold values in the affected ear than in the unaffected ear, eight patients (29.63%) exhibited the same cVEMP threshold values in both ears, and one patient (3.70%) exhibited slightly higher cVEMP threshold values in the unaffected ear than in the affected ear. The cVEMP threshold values of the affected ears were significantly higher than those of the unaffected ears (p = 0.000; Fig 1) and threshold values were elevated by 7.78 ± 8.13 dB nHL.

**Table 2:** Cervical vestibular evoked myogenic potential [cVEMP] and ocular VEMP [oVEMP] response rates of the affected and unaffected ears.

VEMP test	Affected ears		Unaffected ears		Significance [chi-square test]
	N [ears]	Response rates [%]	N [ears]	Response rates [%]	
cVEMP	27	81.48	27	100	c2 = 5.51; p = 0.019
oVEMP	27	51.85	27	96.30	c2 = 13.89; p = 0.000

N, the numbers of ears.

In the oVEMP test, 14 patients (51.85%) exhibited a bilateral response, 13 patients (48.15%) exhibited no response in the affected ear. The response rate of the affected ears was significantly lower than that of the unaffected ears (p = 0.000; Table 2). While one patient exhibited no response in either ear, 18 patients (69.23%) exhibited higher oVEMP threshold values in the affected ear than in the unaffected ear; six patients (23.08%) exhibited the same threshold values in both ears, and two patients (7.69%) exhibited slightly higher oVEMP threshold values in the unaffected ear than in the affected ear. The oVEMP threshold values of the affected ears were significantly higher than that of the unaffected ears (p = 0.001; Fig 1) and threshold values were elevated by 6.67 ± 8.20 dB nHL. The P1 latency, N1 latency, and amplitude in VEMP tests of the affected side and the unaffected side are shown in Table 3.

**Table 3:** Descriptive statistics of the cVEMP and oVEMP tests at 95 dB nHL.

	cVEMP			oVEMP		
	Affected ears	Unaffected ears	P	Affected ears	Unaffected ears	P
N [ears]	22	27		17	26	
P1 latency [ms]	15.33 ± 1.41	16.09 ± 1.80	0.12	15.24±0.47	15.36 ± 0.70	0.57
N1 latency [ms]	23.00 ± 1.92	23.40 ± 2.12	0.47	10.67 ±0.66	10.84 ± 0.80	0.50
Amplitude [μV]	302.18 ± 293.07	385.79 ± 336.32	0.36	26.68 ± 16.90	22.1 ± 14.89	0.38

N, the numbers of ears.

### Variation in the otolith organ function with hearing impairment duration after mumps infection

**Fig 2:**
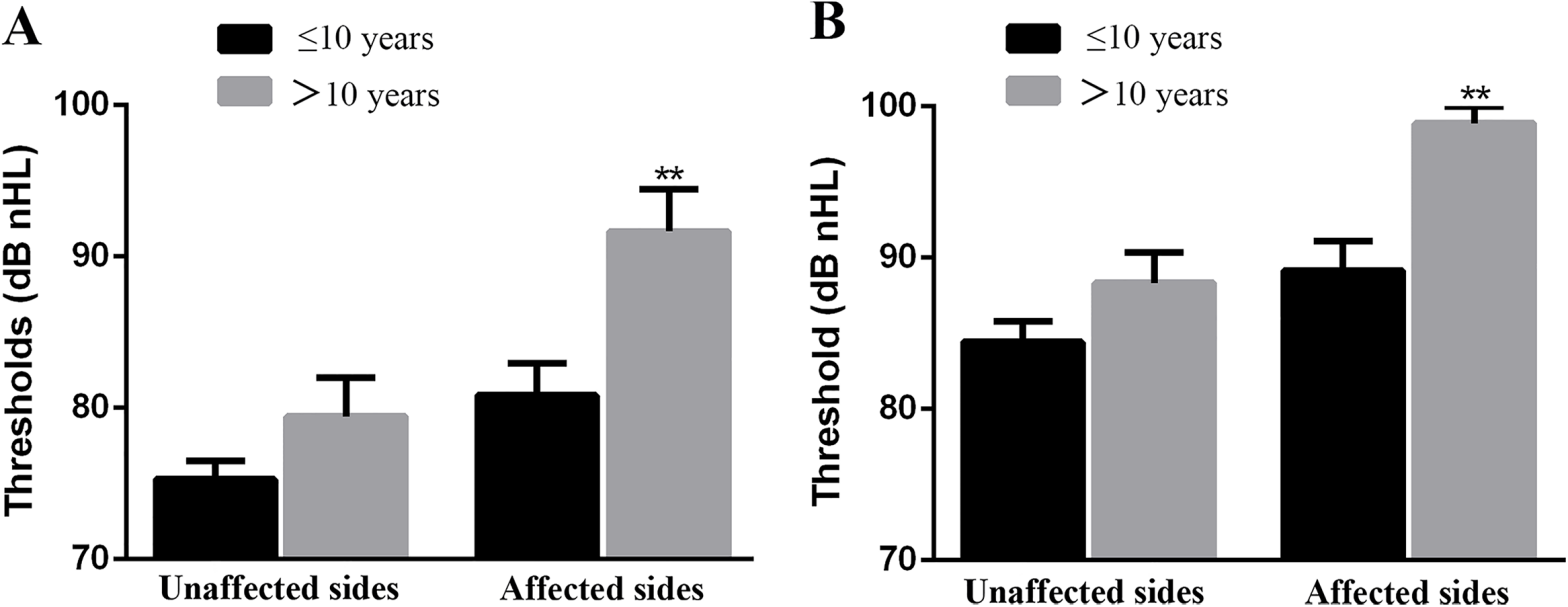
Comparison of mean threshold values of VEMPs between patients with hearing impairment for ≤ 10 years and those with hearing impairment for > 10 years. The columns of the histograms represent the mean values of thresholds and the standard error bars are marked in the histograms. (A)The mean threshold values of cVEMP. (B)The mean threshold values of oVEMP. **p < 0.01. cVEMP, cervical vestibular evoked myogenic potential; oVEMP, ocular vestibular evoked myogenic potential.

In the cVEMP and oVEMP tests, the differences in the mean threshold value of the affected ears between patients with hearing impairment lasting ≤ 10 years and those with hearing impairment lasting > 10 years were statistically significant (p = 0.009 and 0.004 and Fig 2A and 2B, respectively) and the threshold values were elevated by 10.84 dB nHL and 9.72 dB nHL, respectively. In contrast, the differences in the mean threshold values of the unaffected ears between the two groups were not statistically significant in either test (p = 0.18, both; Fig 2A and 2B, respectively).

## Discussion

Although mumps infection results in serious complications, including hearing loss, vestibular dysfunction, aseptic meningitis, and encephalitis, which impose a heavy economic burden to the society and family, studies on hearing loss and otolith organ damage following mumps infection are scarce. In this study, we evaluated the extent of hearing loss and otolith organ dysfunction by audiometry and VEMP tests.

In the present study, the mean age of the patients at the time of mumps infection was 8.56 years, which was similar to the findings of a previous study, which reported that 81.8% of patients with mumps in China were between the ages of 3 and 14 years [2]. However, in a study involving 9663 patients with mumps in the Czech Republic between 2007 and 2012, Orlikova et al. reported that the mean age at disease onset was 17.3 years [19]. This discrepancy in the age at the time of the disease onset among studies may be attributed, among other factors, to the proportion of vaccinated population, degree of economic development, and ethnic and environmental differences among the study populations.

Generally speaking, hearing impairment brought about by mumps is unilateral and irreversible. Profound hearing loss has been reported to occur in 0.05 of 1000 unvaccinated patients [20]. In the present study, all 27 patients exhibited unilateral hearing impairment; while 21 patients exhibited no response upon audiometric examination, the remainder exhibited profound sensorineural hearing loss. These results indicate that mumps infection can cause severe impairment of unilateral hearing. Mumps virus that is present in the blood, cerebrospinal fluid [CSF], or middle ear can infect the inner ear and cause lesions [21]. However, it is not clear whether hearing impairment after mumps is a result of the direct effects of viral infection or the indirect effects of local inflammation caused by the viral infection [9]. Potential mechanisms of mumps-associated deafness include atrophy of hair cells and damage of the vestibulocochlear nerve and brain stem regions [22].

Vaccination can minimize not only the risk of severe health complications, such as profound sensorineural hearing loss, but also of disease transmission among susceptible populations [14]. In order to reduce the burden of mumps on family and society, effective measures, such as extensive vaccination, science education, and research for identification of better treatment, should be undertaken.

In addition to causing sensorineural hearing loss, mumps can also affect the function of otolith organs. The cochlea and vestibule are functionally and anatomically related to each other by means of a shared continuous membranous labyrinth of the inner ear and labyrinthine artery, as well as a similar receptor cell ultrastructure [16]. Therefore, it is reasonable to hypothesize that diseases of the inner ear may influence the function of both the cochlea and otolith organs.

Because of the excessive emphasis on hearing loss, dysfunction of the vestibular system and the related symptoms are easily neglected [16]. After mumps infection, degenerative otoliths have been observed bilaterally in the saccule and utricle, along with a few in the ampullae [23]. In the present study, we found that post-mumps deafness might be accompanied by a lesion of the otolith organs. In addition, the response rates of the affected ears were lower than those of the unaffected ears in both the cVEMP and oVEMP tests. These findings are similar to those reported by El-Badry et al. [15], who showed that 42.1% of patients with post-mumps sensorineural hearing loss exhibited normal VEMP values, while 57.9% exhibited no VEMP response on the affected side. In the present study, we also found that the affected ears exhibited higher threshold values of cVEMP and oVEMP than did the unaffected ears, which further illustrates that mumps could certainly cause lesions in the otolith organs of the affected ears. However, only one patient suffered from vertigo and exhibited mild abnormalities in the caloric test and sensory organization test. The other 26 patients had no complaints of dizziness and imbalance, or any symptom of vestibular dysfunction. This may be due to the following reasons: 1) The damage in the otolith organs is too mild to cause symptoms, and is thus hidden damage. 2) Unilateral otolith organ damage could be compensated for by the vestibular central system. 3) The damage is progressive, and visual sensory systems and proprioceptive sensory systems can compensate for the dysfunction of otolith organs. Otolithic dysfunction can cause a variety of serious problems, including falling down, imbalance, dizziness, spatial disorientation, and blurring of vision. It is often obscured by clinical symptoms, such as hearing loss. If the damage to the otolith organs could be detected early and appropriate intervention implemented, these severe symptoms which affect the quality of life may not occur. Therefore, encourage doctors to pay more attention to the occult damage to the otolith organs. In addition, the mean threshold values of VEMPs in the affected ears exhibited significant increments with the time elapsed after mumps infection. This suggests that the function of otolith organs or their neural pathway may suffer secondary damage over time.

## Conclusion

In the present study, we have investigated the extent and possible mechanisms of deafness and otolith organ dysfunction after mumps infection. Mumps, a disease that usually affects the salivary glands, is the most common cause of unilateral acquired sensorineural hearing loss, especially in children. Deafness caused by mumps is usually profound and permanent. Atrophy of hair cells have been observed [22], indicating severe damage to the cochlea as a result of virus infection. The effect of mumps infection on the function of otolith organs in the vestibular system has rarely been studied. Our findings indicate that otolith organ dysfunction should also be considered as one of the major concerns after mumps infection, especially in patients exhibiting profound hearing loss. The pathogenesis and pathological changes of otolith organ dysfunction after mumps infection should be further explored in future studies.

## Supporting information

S1 File The electrode montage of cVEMP and oVEMP. (PDF)
