# Supplementary material for: The potential dysfunction of otolith organs in patients after mumps infection
Source: PLoS One. 2017 Jul 26;12(7):e0181907. doi: 10.1371/journal.pone.0181907 (PMC5528881; doi:10.1371/journal.pone.0181907)
Supplement: S1 File — (PDF) [file pone.0181907.s001.pdf]

1. The electrode montage of cVEMP（The related information of AEP User and Service Manual ， Page24）.

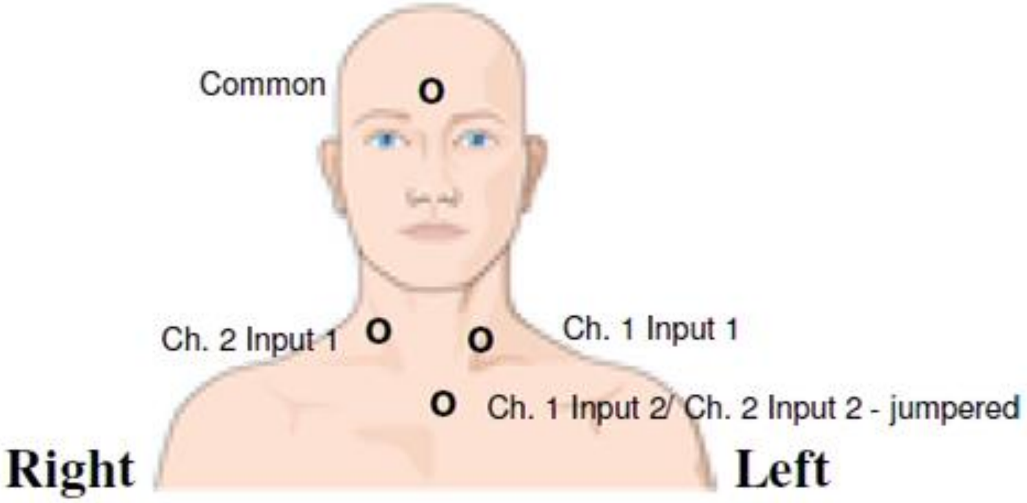

Left sound stimulation: Active (channel 1, input 1); Reference (channel 1, input 2); Common(Ground).

Right sound stimulation: Active (channel 2, input 1); Reference (channel 2, input 2); Common(Ground).

Description: An active electrode on the middle of the sternocleidomastoid muscle (SCM), with a reference electrode on the lower part of the suprasternal fossa and a ground reference on the middle of the forehead.

2. The electrode montage of oVEMP.

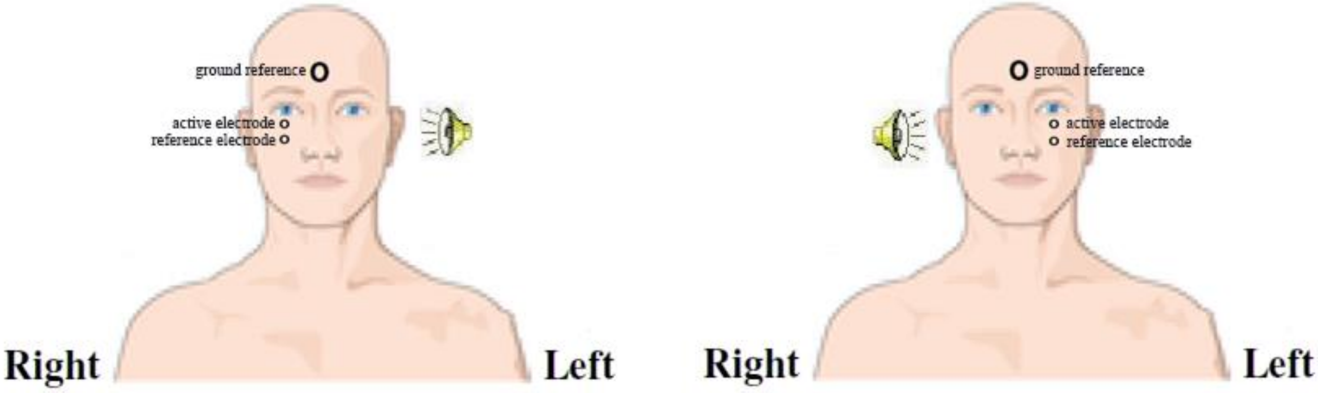

Description: An active electrode was placed about 1 cm below the center of the inferior eyelid of the right eye contrary to the side of sound stimulation, with a reference electrode 2cm below the active electrode and a ground reference on the middle of the forehead.
